# Supplementary material for: Effectiveness and Safety of Ayurvedic Medicines in Type 2 Diabetes Mellitus Management: A Systematic Review and Meta-Analysis
Source: Front Pharmacol. 2022 Jun 8;13:821810. doi: 10.3389/fphar.2022.821810 (PMC9213670; doi:10.3389/fphar.2022.821810)
Supplement: Supplementary file 1 [file DataSheet1.zip › Supplementary Material/Forest and Funnel Plots/Portulaca oleracea/FBG.pdf]

Portulaca oleracea - FBG

Study	Intervention N	Intervention Mean	Intervention SD	Comparator N	Comparator Mean	Comparator SD	Mean Diff. with 95% CI	Weight (%)
Farzanegi 2014	14	131.60	17.93	14	159.35	40.11	-27.76 [-50.77, -4.74]	37.10
Dehghan 2016	16	146.10	37.81	16	160.20	53.63	-14.10 [-46.25, 18.05]	27.98
Wainstein 2016	31	181.70	52.20	32	170.60	49.10	11.10 [-13.92, 36.12]	34.93
**Overall**							-10.36 [-34.32, 13.59]	

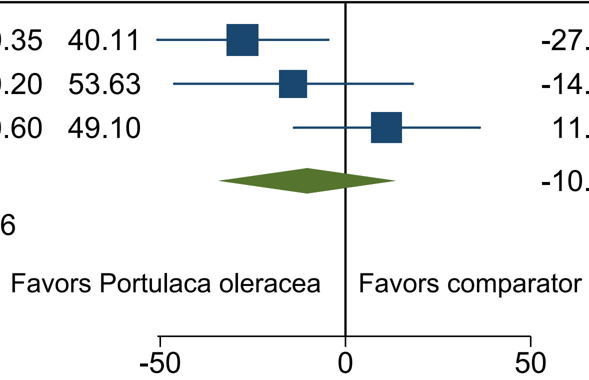

Heterogeneity: $\tau^2 = 264.80$, $I^2 = 59.34\%$, $H^2 = 2.46$

Test of $\theta_i = \theta_j$: Q(2) = 5.07, p = 0.08

Test of $\theta = 0$: z = -0.85, p = 0.40

Random-effects REML model
